# A de novo variant in *ZBTB18* gene caused autosomal dominant non-syndromic intellectual disability 22 syndrome: A case report and literature review

**DOI:** 10.1097/MD.0000000000035908

**Published:** 2024-01-12

**Authors:** Fan Yang, Yu Ding, Yirou Wang, Qingwen Zhang, Hao Li, Tingting Yu, Guoying Chang, Xiumin Wang

**Affiliations:** a Clinical Research Ward, Shanghai Children’s Medical Center, Shanghai Jiao Tong University School of Medicine, Shanghai, China; b Department of Endocrinology and Metabolism, Shanghai Children’s Medical Center, Shanghai Jiao Tong University School of Medicine, Shanghai, China; c Department of Pharmacy, Shanghai Children’s Medical Center, Shanghai Jiao Tong University School of Medicine, Shanghai, China; d Department of Medical Genetics and Molecular Diagnostic Laboratory, Shanghai Children’s Medical Center, Shanghai Jiao Tong University School of Medicine, Shanghai, China

**Keywords:** autosomal dominant intellectual disability, C2H2 zinc finger, intellectual disability, *ZBTB18* gene

## Abstract

**Rationale:**

Autosomal dominant non-syndromic intellectual disability 22 is a rare genetic disorder caused by the *ZBTB18* gene. This disorder affects various parts of the body, leading to intellectual disability. It is noteworthy that only 31 cases of this disorder have been reported thus far. As the symptom severity may differ, doctors may face challenges in diagnosing it accurately. It is crucial to be familiar with this disorder’s symptoms to receive proper diagnosis and essential medical care.

**Patient concerns:**

There is a case report of a 6-year-old boy who had an unexplained thyroid abnormality, global developmental delay, and an abnormal signal of white matter in brain MRI. However, he did not have growth retardation, microcephaly, corpus callosum hypoplasia, epilepsy, or dysmorphic facial features. Clinical whole exome sequencing revealed a de novo pathogenic variant in the *ZBTB18* gene (c.1207delC, p. Arg403Alafs*60), which is a previously unreported site. This variant causes the premature termination of peptide chain synthesis, leading to incomplete polypeptide chains.

**Diagnoses:**

Autosomal dominant non-syndromic intellectual and disability 22 syndrome and thyroid dysfunction.

**Interventions:**

Rehabilitation training.

**Outcomes:**

The individual is experiencing difficulty with their motor skills, appearing clumsier while running. He struggles with expressing themselves and forming complete sentences, relying mostly on gestures and pointing.

**Lessons:**

The clinical presentations of mental retardation, autosomal dominant, type 22 (MRD22) are complicated and varied. Although early diagnosis can be made according to typical clinical symptoms, whole exome sequencing is necessary for diagnosing MRD22, as our study indicates.

## 1. Introduction

Autosomal dominant non-syndromic intellectual disability 22 is also known as mental retardation, autosomal dominant, type 22 (MRD22). MRD22 syndrome (OMIM #612337) is characterized by intellectual disability (ID), growth retardation, microcephaly, corpus callosum (CC) hypoplasia, epilepsy, and dysmorphic facial features (prominent forehead, hypertelorism, flat nasal bridge, epicanthal folds, round face, and malformed low-set ears).^[1]^ It was found that the heterozygous variant in *ZBTB18* gene was the main cause of the disease.

The *ZBTB18* gene (OMIM# 608433), formerly known as RP58, ZFP238, or ZNF238,^[2]^ is located on 1q44, encodes ZBTB18 (zinc finger and BTB domain-containing protein 18, ZBTB18), which acts as a transcriptional repressor of key proneuronal genes^[3]^ and includes an N-terminal BTB domain for protein interaction and 4 C-terminal zinc finger proteins for DNA binding.^[4]^ Patients with 1q43q44 microdeletion syndrome (OMIM#612337) present overlapping phenotypes with MRD22 syndrome.^[5–7]^ The complete neurodevelopmental phenotype of patients with 1q43q44 microdeletion syndrome is the result of deletions of 3 major genes (*AKT3, ZBTB18*, and *HNRNPU*) spanning 1.36 Mb.^[8]^

Currently, only 31 cases of *ZBTB18* gene mutation have been reported in the literature. Here we report a patient who carries a de novo pathogenic variant (c.1207delC, p.Arg403Alafs*60) in *ZBTB18* gene by whole exome sequencing (WES) and review the relevant literature to promote clinical diagnosis and treatment. We present the following article in accordance with the CARE reporting checklist.

## 2. Clinical reports

### 2.1. General information

**Figure 1.**
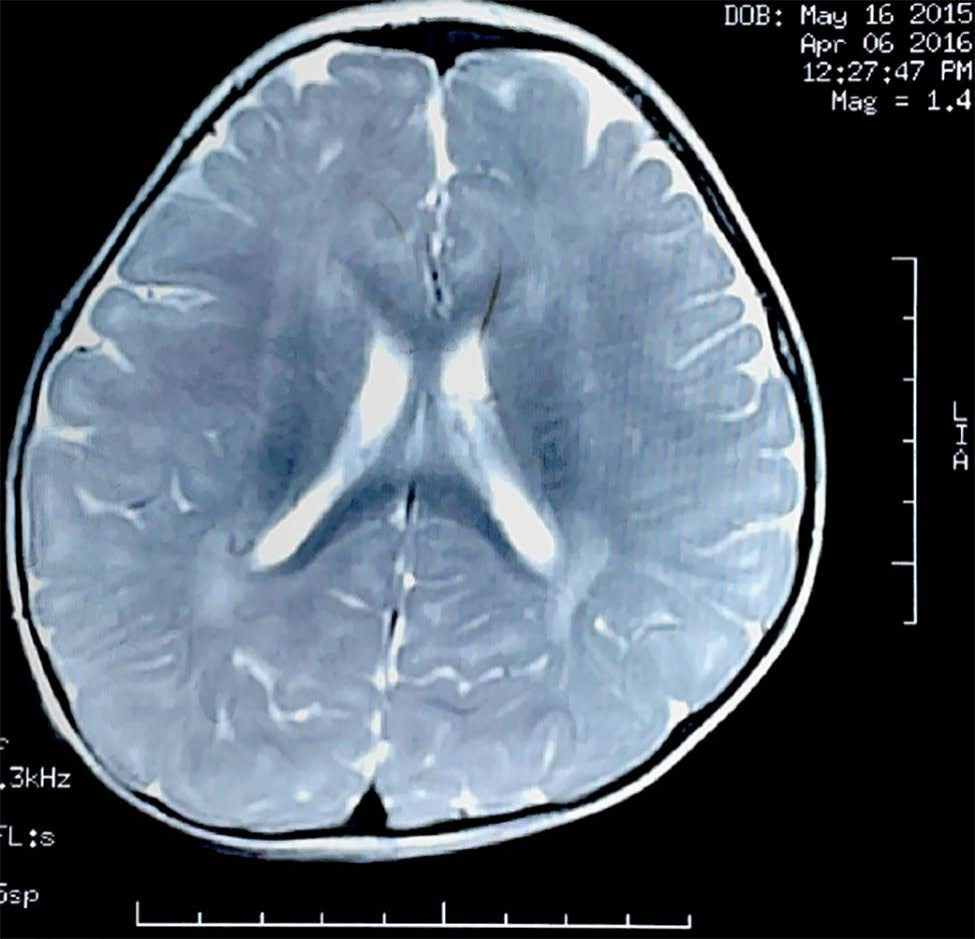
MRI images. Patient at age 11 months: abnormal signal in the white matter around the posterior part of the bilateral lateral ventricles.

The proband is the second child born to a non-consanguineous couple of Chinese ancestry with no notable family history. His older sister is developing normally, healthy, and has a normal body size. He was born at term following an uneventful pregnancy with a birthweight of 3.3 kg (25th–50th centile), birth length of 50 cm (25^th^–50th centile), and head circumference of 34 cm (25^th^–50th centile). From a motor standpoint, he first rolled at 11 months, sat alone at 13 months, crawled at 15 months, and walked with support at 18 months. He had significant language delay in that he did not speak his first words until 13 months and did not put together two-to-three words sentences until 18 months. He was globally delayed in development since birth, his brain magnetic resonance imaging (MRI) showed abnormalities in the posterior periventricular white matter (Fig. 1). At present, he was able to run but was clumsier. He can neither verbalize a complete sentence nor pronounce words clearly. He has a few signs but mostly gestures and points. He is able to follow one step commands. He gets frustrated with his inability to communicate at times.

**Figure 2.**
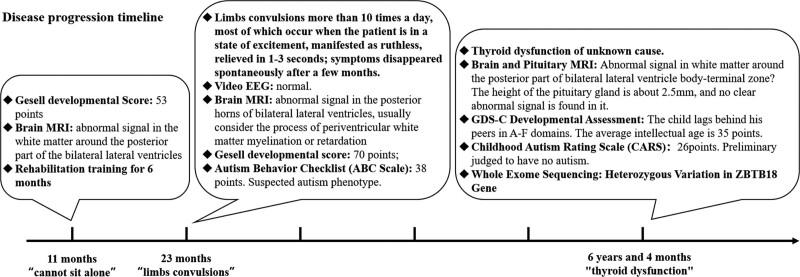
The disease progression timeline of the patient. At 11 months of age, he was found globally development delayed and had abnormalities brain MRI. At the age of 23 months, He developed limb shaking with normal electroencephalography (EEG) and resolved spontaneously after several months. At 6 years and 4 months of age, he was first visited a genetics clinic for an unexplained thyroid abnormality.

He was first visited a genetics clinic at 6 years and 4 months of age for global developmental delay and an unexplained thyroid abnormality: Thyroid indicators showed hyperfunction but no obvious symptoms, the local hospital did not identify the cause of thyroid dysfunction. He was admitted to our hospital and took WES. His physical examination at that age were: length 119.6 cm (50^th^–75th), weight 20.9 kg (25th–50th), head circumference 52 cm (normal), heart rate 102 beats per minute, with non-moist skin, no rash, no exophthalmos, no facial dysmorphism, no thyroid enlargement, stable breathing, and the muscle strength and tension of the limbs were normal. Uncooperative tremor checks for both hands, while the rest of the physical examination was unremarkable. Laboratory tests in other hospitals: thyroid antibodies (A-TPO, A-TG, TG, TRAb), glycosylated hemoglobin, blood glucose, insulin, C-peptide, adrenocorticotropic hormone, cortisol, liver and kidney function, electrolytes, myocardial enzymes, blood lipids, immunity index (anti-nuclear antibody spectrum, anti-cardiolipin antibodies, complement C3, C4), based serum hormone and thyroid ultrasound were all normal. The Griffiths Development Scales-Chinese Edition reported the patient’s intelligence quotient score as 35, which indicated that he is severe ID (Fig. 2).

### 2.2. Genetic analysis

#### 2.2.1. DNA extraction.

After obtaining the informed consent of the patient’s parents and signing the informed consent form, 2 mL of peripheral intravenous EDTA anticoagulated whole blood was drawn from the patient and his parents, and the genome was extracted using the QIAamp Blood DNA Mini kit kit (Qiagen GmbH, Hilden, Germany). DNA, using a NanoDrop2000 spectrophotometer (Thermo Scientific, USA) to measure the concentration of genomic DNA and store at −20°C.

#### 2.2.2. Whole exome sequencing.

Take 3 μg of genomic DNA, use Covarias M220 Ultrasonicator system (Covaris, Inc. Woburn, MA) to break the genomic DNA into fragments of 150 to 200 bp. The sequencing library was constructed using SureSelect XT Human All Exon V6 kit (Agilent Technologies, Inc., Santa Clara, CA), and then high-throughput sequencing was performed on the Illumina NovaSeq 6000 platform.

#### 2.2.3. Data analysis.

The sequencing raw data in Fastq format were processed by NextGene software (SoftGenetics, USA) and aligned with the reference sequence (GRCh37/hg19). All variants were saved in VCF format files and uploaded to Ingenious online software (Qiagen, Germany) for biological analysis and annotation. After removing low-confidence variants, the following analysis strategies were used to further filter variants: remove variants with a frequency >1% in the gnomAD (http://gnomad.broadinstitute.org/) database; remove missense variants that are predicted to be harmless by SIFT, Polyphen2, MutationTaster software; remove synonymous variants that are predicted to not cause splicing abnormalities by MaxEnt, NNSPLICE software. The finally identified candidate variants consistent with the clinical phenotype were verified in the patient and his parents by first-generation Sanger sequencing technology, and the possible pathogenic copy-number variants were excluded through deep sequencing analysis.

### 2.3. Statistical analysis

The collected data were analyzed using SPSS software (v. 22.0, SPSS Inc., Chicago, IL). The main statistical analyses are descriptive. Categorical data were expressed as number and percentage (%) and Chi square or Fisher exact test were used for categorical variables. A 2-sided *P* value less than .05 was considered as statistically significant.

## 3. Results

### 3.1. High-throughput sequencing results, Sanger sequencing verification

**Figure 3.**
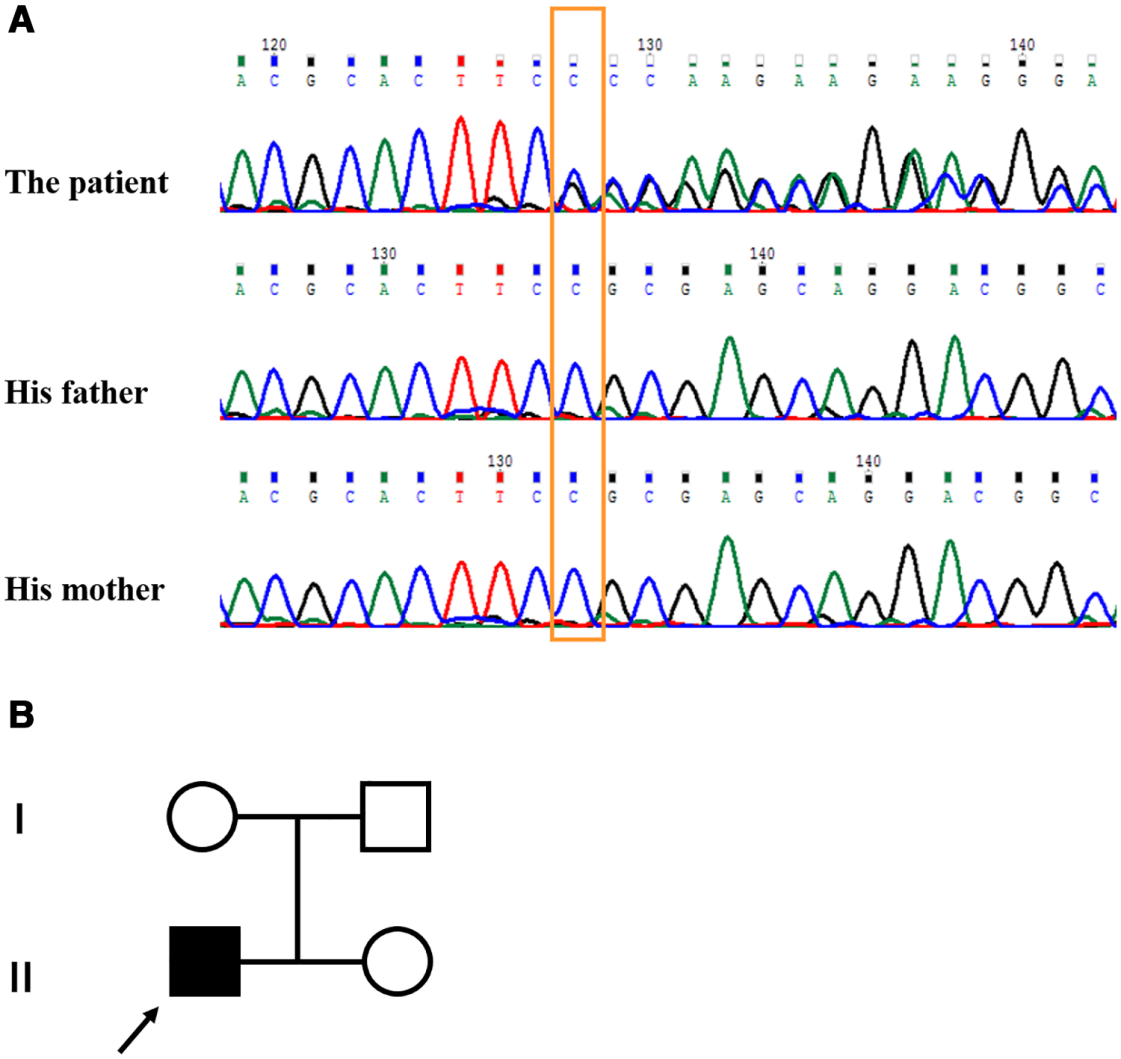
(A) Novel mutation was identified in the *ZBTB18* gene. Results of Sanger sequencing of *ZBTB18* (NM_205768) showing de novo occurrence of the mutation c.1207delC (p.Arg403Alafs*60). (B) The family tree.

Whole-exome sequencing showed that the patient had a heterozygous variant in the *ZBTB18* gene (NM_205768.3): a base deletion c.1207delC (p.Arg403Alafs*60) in exon 1 (Fig. 3A). Its parental *ZBTB18* gene is wild type (Fig. 3B). This variant is newly discovered (not included in databases such as HGMD and gnomAD), which can cause premature termination of amino acid translation or affect mRNA expression. According to the ACMG variant classification criteria, it can be classified as “pathogenic” variants.

### 3.2. Analysis of *ZBTB18* variants and clinical spectrum

**Figure 4.**
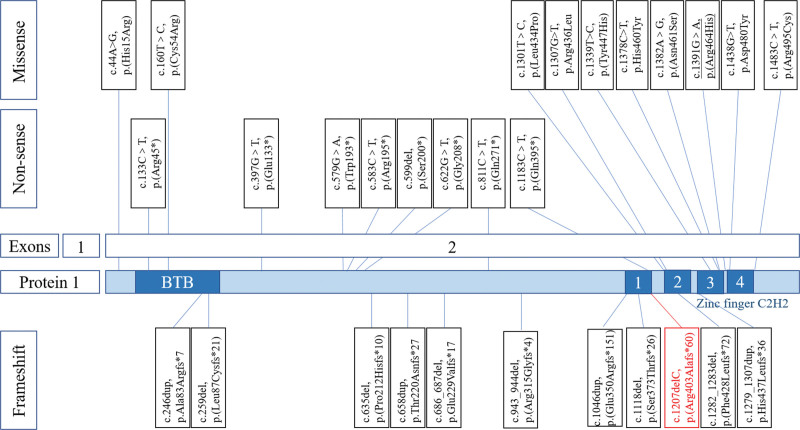
Summary of intragenic microdeletions and point mutations in *ZBTB18* (reference transcript NM_205768.2). Including this study (red frame) and the literature (black frame). The underlined site (p.Arg464His) was found in 4 reported patients.

We provide a complete overview of the 32 *ZBTB18* pathogenic variants reported to date, including our case, showing the inconsistent presence of clinical features (Table 1). A significant proportion of *ZBTB18* variants were missense variants (40.6% (13/32), 34.3% (11/32) were frameshift and 25% (8/32) were nonsense). Notably, the vast majority (84.6%; 11 of 13) of *ZBTB18* missense variants are located within the C-terminal zinc-finger DNA-binding region, which is critical for its role in transcriptional regulation important (Fig. 4).

**Table 1:** Clinical phenotype and genotype statistics of 32 patients.

Clinical symptoms	Total* (n = 32)	Missense (n = 13) n (%)	Nonsense (n = 8) N (%)	Frameshift (n = 11) n (%)
Growth				
Postnatal growth delay	5/23 (21.7%), 9 NA	0 (0)	2 (33.3)	3 (33.3)
Microcephaly	9/27 (33.3%), 5NA	2 (22.2)	4 (66.7)	1 (14.3)
Development				
Intellectual disability	32/32 (100%)	13 (100)	8 (100)	11 (100)
Speech delay	30/31 (96.8%), 1NA	12 (100)	8 (100)	10 (90.9)
Neurological and other findings				
Corpus callosum hypoplasia	10/17 (58.8%), 15NA	5 (71.4)	4 (66.7)	1 (25)
Epilepsy	6/17 (35.3%), 15NA	3 (37.5)	2 (40)	1 (25)
Ataxia/hypotonia	11/19 (57.9%), 13NA	6 (66.7)	3 (50)	2 (50)
Congenital anomalies				
Facial features†	15/25 (60%), 7NA	5 (83.3)	4 (80)	4 (44.4)
Other anomalies‡	4/20 (19.0),12NA	2 (33.3)	1 (14.3)	1 (14.3)

NA = not applicable.

* Summary of key features (n/total with information available)

† Facial features are variable but characteristic, including round face, prominent forehead, flat nose bridge, distant eyes, epicanthus, and low-set ears.

‡ Other anomalies including cardiac, urogenital and gastrointestinal.

We summarized the clinical phenotypes and found that almost all patients had some degree of intellectual disability (32 of 32, 100%) and language retardation (30/31, 96.8%), and the vast majority of children had dysmorphic facial features (15/25, 60%), 58.8% (10/17) had corpus callosum abnormalities, 57.9% (11/19) had ataxia, 35.5% (6/17) had epilepsy, 33.3% (9/27) had microcephaly, and 21.7% (5/23) had growth retardation. The clinical phenotypes of missense variants, nonsense variants, and frameshift variants were not statistically different. Our data do not support a clear genotype-phenotype correlation.

### 3.3. Treatments

The patient took methimazole orally for a few weeks, thyroid function improved during follow-up, and methimazole was gradually discontinued. The thyroid function of the children was continuously monitored in the local hospital for 1 year after drug withdrawal. TSH was elevated, FT3 was high or normal, and FT4, T3 and T4 were normal. Thyroid antibody tests were negative multiple times. According to the clinical manifestation, laboratory examination, and ZBTB18 gene mutation site, he was diagnosed as MDR22 and thyroid dysfunction.

## 4. Discussion

In this report, we presented a novel pathogenic variant in the *ZBTB1*8 *gene that causes MRD22 syndrome by WES.* The proband’s clinical presentations of intellectual disability, delayed speech, and abnormal brain MRI were similar with those previously reported cases in MRD22 syndrome or with a deletion of 1q43q44.^[9]^ Based on our review of 9 articles retrieved from PubMed, 31 cases of MRD22 caused by *ZBTB1*8 variants are summarized in Table 2. There were 29 pathogenic variants of *ZBTB18* gene, including 4 cases with C.1391G>A (p.Arg464His). Among the known *ZBTB18* variants, there were 13 missense variants, 11 frameshift variants, 8 nonsense variants. We describe a novel variant in *ZBTB18* gene c.1207delC (p.Arg403Alafs*60), which is a frameshift variant. The type of gene mutation in this case enriched the *ZBTB18* gene profile database of 1q43q44 deletion syndrome and is conducive to clinical genetic counseling.

**Table 2:** Summary of clinical phenotypes of patients with *ZBTB18* gene variants.

	This study	Rauch et al (2012)	De Munnik et al (2014)	Lopes et al (2016)	McRae et al (2016)					Cohen et al (2017)							Depienne et al (2017)				Van der Schoot et al (2018)				Wang et al (2020)							Zhang et al (2022)	Summary of Key Features*
	1	2	3	4	5	6	7	8	9	10	11	12	13	14	15	16	17	18	19	20	21	22	23	24	25	26	27	28	29	30	31	32	
	p.(Arg403Alafs*60)	p.(Arg-495Cys)	p.(Glu-133*)	p.(Arg-195*)	p.(Gly-208*)	p.(Pro-212Hisfs*10)	p.(Gln-271*)	p.(Glu-350Argfs*15)	p.(Arg-464His)	p.(Ans-461Ser)	p.(Arg-315Glyfs*4)	p.(Gln-395*)	p.(Arg-464His)	p.(Arg-45*)	p.(Ser-373Thrfs*26)	p.(Cys-54Arg)	**p.(His-15Arg**)	**p.(Arg-464His**)	**p.(Leu-434Pro**)	p.(Ser-200*)	**p.(Tyr-447His**)	p.(Trp-193*)	p.(Leu-87Cysfs*21)	**p.(Arg-464His**)	p.(Glu229Valfs*17)	p.(Thr220Asnfs*27)	p.(Ala83Argfs*7)	p.(His437Leufs*36)	**p.(Arg436Leu**)	**p.(His460Tyr**)	**p.(Asp480Tyr**)	p.(Phe428Leufs*72)	
Gender	M	F	F	F	M	F	M	M	F	M	M	M	M	M	M	NA	M	F	M	F	M	M	M	M	M	M	M	M	F	F	F	Male	
Age at examination (yr)		18	2.8	5	Not reported (Individual values not reported)					7	3	4	34	6	15	NA	14	12	23	12	15	1	13	4	7	12	17	18	6	1	3.7	3.7	4
Postnatal growth delay	–	–	+	–	5NA					–	–	+	–	–	–	NA	–	–	–	–	–	–	–	–	2/4 (50%) (Individual values not reported)				NA	NA	NA	+	5/23(21.7%), 9 NA
Microcephaly	–	–	+	+	2/5 (40%)					–	–	+	–	–	–	+	–	–	–	–	+	+	–	–					NA	NA	NA	–	9/27 (33.3%), 5 NA
Intellectual disability	+	+	+	+	+	+	+	+	+	+	+	+	+	+	+	+	+	+	+	+	+	+	+	+					+	+	+	+	32/32 (100%)
Speech delay	+	+	+	+	+	+	+	+	+	+	+	+	+	+	+	+	+	+	+	+	+	+	+	+					+	+	NA	+	30/31 (96.8%), 1 NA
Corpus callosum hypoplasia	–	–	–	–	5NA					+	+	+	NA	+	NA	NA	+	+	NA	+	–	+	–	+					NA	NA	+	–	10/17 (58.8%),15 NA
Epilepsy	–	NA	–	NA	5NA					–	–	–	–	+	NA	NA	–	–	+	+	+	–	–	–					NA	+	NA	+	6/17 (35.3%), 15 NA
Ataxia/hypotonia	+	NA	–	+	5NA					+	+	+	–	+	NA	NA	+	–	–	–	+	–	–	+					NA	+	+	–	11/19 (57.9%),13 NA
**Facial features** †	–	NA	+	–	2/5 (40%)					–	+	+	+	+	–	+	NA	NA	NA	NA	+	+	+	+					+	NA	NA	–	15/25 (60%), 7 NA
**Other anomalies** ‡	–	NA	–	–	–					+	–	+	–	–	–	NA	NA	NA	NA	NA	–	–	+	+					–	NA	NA	–	4/20 (19.0),12 NA
Others	Thyroid dysfunction, Periventricular white matter abnormalities		Gastro-intestinal problems	bruxism	2/5 with finger, 3/5 with hair abnormalities					Myopia	Strabismus	Strabismus, single febrile seizure	Drooling																	visual impairment, hypsarrhythmia	brother with developmental delay and hypotonia.		

F = female, M = male, “+” = positive, “–” = negative, “NA” = not applicable.

* Summary of key features (n/total with information available).

† Facial features are variable but characteristic, including round face, prominent forehead, flat nose bridge, distant eyes, epicanthus, and low-set ears.

‡ Other anomalies including cardiac, urogenital and gastrointestinal.

*ZBTB18* encodes a protein with 4 Cys2His2 zinc-fingers (C2H2-ZNF) for DNA binding, which plays a central role in gene regulation and cellular function.^[10]^ Interactions with other protein partners through its BTB domain, including DNMT3A, facilitate the gene repressive activity of *ZBTB18*. Impaired binding of *ZBTB18* to DNA interferes with its function as a transcriptional repressor.^[11]^ *ZBTB18* has high sequence conservation among vertebrate orthologues, with remarkably similar amino acid composition within the DNA binding zinc finger domain.^[11]^ For example, total human protein identity is 99.4% with *M musculus* and 78% with *Danio rerio*, with even higher identity in the C2H2 ZNF domain.^[12]^ Such sequence conservation within this domain can in part be explained by its importance for transcriptional regulation. In humans, for example, *ZBTB18* missense variants that are associated with brain developmental disease overwhelmingly map to the zinc finger domain.^[11]^ We consider variants in the C2H2 region of other genes (such as *ZBTB20* and *YY1*) that also cause phenotypic features by disabling appropriate DNA binding. Our research found a significant proportion of *ZBTB18* variants were missense variants (40.6%, 13/32), and the vast majority (>84.6%; 11 of 13) were located within the C-terminal zinc-finger DNA-binding region. Hemming et al^[4]^ investigated studied *ZBTB18* as a protein-DNA complex and found that most disease-associated missense variants map to DNA-contact residues essential for motif binding and can affect *ZBTB18* function, with implications for neurodevelopment, homeostasis, and disease. Nonsense and frameshift variants lead to premature stop codons in the last (second) exon of the *ZBTB18* gene. These lesions result in the loss of DNA-binding domains, and the expected truncated proteins may become dysfunctional. Zhang et al^[13]^ found that in the clinical phenotype of *ZBTB18* gene, frameshift/nonsense variants of the gene may be more prone to ID and speech delay than missense variants. Patients collected by Zhang et al^[13]^ had mutations in 1q43q44 deletion. However, our research counted patients only with *ZBTB18* mutations, and we did not find a genotype-clinical phenotype correlation.

Global developmental delay/ID is in all 32 patients (100%). Neurological symptoms echo the findings in animal experiments.^[6]^ The incidence of short stature is as high as 21.7% (5/23). Some children develop growth retardation in utero, often manifested as low birth weight. About 33.3% (9/27) of them had head circumference less than -2SD below the mean of children of the same age and sex.^[5,14]^ CC abnormalities were evident in 58.8% (10/17) patients who underwent neuroimaging. In addition to CC, Cohen et al^[6]^ and Depienne et al^[8]^ reported abnormal MRI findings also including pituitary abnormalities, small hippocampal disorders, and cerebellar vermis hypoplasia. The white matter of the brain contains nerve tracts with different functions, among which CC contains the largest and most homogeneous fiber tracts in the human brain.^[15]^ We report the first case of a patient with abnormal cranial MRI (white matter signal abnormal) but normal CC. Ataxia or hypotonia have been reported in 57.9% (11/19) of MRD22 children, Epilepsy have been reported in 35.3% (6/17), and a small number of children are accompanied by autism, ADHD, and behavioral problems.^[2,8]^

The pathogenesis of ID caused by functional deficiency of *ZBTB18* is unclear, it may be involved to multiple functions of *ZBTB18* in brain development. Mouse with the full knockout of *ZBTB18* is embryonically lethal, while conditional knockout (cKO) shows CC hypoplasia, cerebellar hypoplasia,^[3,16]^ *ZBTB18* can also affect the growth and development of the cerebellum by regulating the transcription of neuronal progenitor cells and affecting the proliferation and differentiation of cerebellar GABAergic neurons.^[3]^ Ohtaka et al found that *ZBTB18* expression were detected in cerebral cortex, part of the amygdala, and cerebellum, and at high levels during formation of the hippocampus. Northern blot analysis has detected *ZBTB18* transcripts in a variety of tissue types, including brain, skeletal muscle, pancreas, testis, and spleen.^[16]^ The importance of time- and place-specific expressions is hypothetical.^[14]^ Haploinsufficiency may be the pathogenic mechanism of *ZBTB18* variants. *ZBTB18* represses PAX6 (OMIM# 607108), NEUROG2 (OMIM# 606624), and NEUROD1 (OMIM# 601724).^[14]^ The expression of these 3 consecutive proneuronogenic genes leads to the differentiation and migration of intermediate neurogenic progenitors (INPs).^[3]^ *ZBTB18* is highly expressed in the cortex and involved in the formation of the Ngn2-Rnd2 pathway which is closely related to the migration of cortical neurons,^[3]^ which may help explain the phenotypic differences between patients.

Dysmorphic facial features including prominent forehead, hypertelorism, flat nasal bridge, epicanthal folds, round face, and malformed low-set ears.^[6]^ 60% (15/25) of the cases are present with facial dysmorphism and 19% (4/20) with other congenital anomalies. Notably, eye diseases (myopia, strabismus and visual impairment) were present in 4 cases,^[2,6]^ we speculate that attention should be paid to ocular features in children with MRD22.

Multiple patient series have been published to describe the clinical phenotype of *ZBTB18*-related disease, such as gastrointestinal problems, bruxism, clinodactyly of 5th finger, abnormality of hair or hair pattern, cerebellar tonsillar hypoplasia, febrile seizure, Myopia, Strabismus, Drooling, stereotype, visual impairment, and other clinical manifestations (Table 2).^[6,17,18]^ The presentation of thyroid dysfunction in our patient has not been previously reported.^[6,14]^ It is still controversial whether the abnormal thyroid function during the follow-up is an experimental error of the testing institution. Recent studies have shown that *ZBTB18* inhibits the expression of PI3K,^[12]^ which plays a role in reducing thyroxine levels and even thyroid cancer.^[19]^ However, there is no relevant research on the activation of PI3K/AKt pathway by *ZBTB18* gene mutation leading to thyroid disease. Notably, data reported by McRae et al^[18]^ and Wang et al^[2]^ included only limited clinical information with no detailed description of individual patients. It will be interesting to see if thyroid dysfunction is a feature of other patients harboring a *ZBTB18* defect.

In conclusion, the main manifestations of MRD22 caused by *ZBTB18* gene variants are intellectual disability (ID), growth retardation, microcephaly, corpus callosum (CC) hypoplasia, epilepsy and dysmorphic facial features, etc. At present, the diagnosis of MRD22 mainly relies on genetic testing. For children with the above clinical manifestations, it is necessary to be alert to the disease. Early genetic testing is helpful for the diagnosis of the disease and provides a basis for further clinical guidance and genetic counseling.

## Acknowledgments

The authors would like to thank the patient and his parents for allowing us to publish this case report.

## Author contributions

**Conceptualization:** Fan Yang, Xiumin Wang.

**Data curation:** Fan Yang, Yu Ding, Xiumin Wang.

**Formal analysis:** Fan Yang, Qingwen Zhang, Tingting Yu.

**Funding acquisition:** Guoying Chang.

**Investigation:** Tingting Yu.

**Methodology:** Qingwen Zhang.

**Writing—original draft:** Fan Yang.

**Writing—review & editing:** Yu Ding, Yirou Wang, Hao Li, Guoying Chang, Xiumin Wang.

## References

[1] Lloveras E Canellas A Barranco L. A new case with corpus callosum abnormalities, microcephaly and seizures associated with a 23-Mb 1q43-q44 Deletion. Cytogenet Genome Res. 2019;159:126–9. 31830750 10.1159/000504424

[2] Wang T Hoekzema K Vecchio D. Large-scale targeted sequencing identifies risk genes for neurodevelopmental disorders. Nat Commun. 2020;11:4932. 33004838 10.1038/s41467-020-18723-y PMC7530681

[3] Xiang C Frietze KK Bi Y. RP58 represses transcriptional programs linked to nonneuronal cell identity and glioblastoma subtypes in developing neurons. Mol Cell Biol. 2021;41:e0052620. 33903225 10.1128/MCB.00526-20 PMC8315738

[4] Hemming IA Blake S Agostino M. General population ZBTB18 missense variants influence DNA binding and transcriptional regulation. Hum Mutat. 2020;41:1629–44. 32598555 10.1002/humu.24069

[5] Hemming IA Clement O Gladwyn-Ng IE. Disease-associated missense variants in ZBTB18 disrupt DNA binding and impair the development of neurons within the embryonic cerebral cortex. Hum Mutat. 2019;40:1841–55. 31112317 10.1002/humu.23803

[6] Cohen JS Srivastava S Farwell Hagman KD. Further evidence that de novo missense and truncating variants in ZBTB18 cause intellectual disability with variable features. Clin Genet. 2017;91:697–707. 27598823 10.1111/cge.12861

[7] Ehmke N Karge S Buchmann J. A de novo nonsense mutation in ZBTB18 plus a de novo 15q133 microdeletion in a 6-year-old female. Am J Med Genet A. 2017;173:1251–6. 28345786 10.1002/ajmg.a.38145

[8] Depienne C Nava C Keren B.; DDD Study. Genetic and phenotypic dissection of 1q43q44 microdeletion syndrome and neurodevelopmental phenotypes associated with mutations in ZBTB18 and HNRNPU. Hum Genet. 2017;136:463–79. 28283832 10.1007/s00439-017-1772-0 PMC5360844

[9] Birnbaum R Markovitch O Biron-Shental T. Prenatal diagnosis of a likely pathogenic variant in ZBTB18: Natural evolution of fetal phenotype including the long bones and corpus callosum. Am J Med Genet A. 2022;188:978–83. 34907638 10.1002/ajmg.a.62599

[10] Aizenshtein-Gazit S Orenstein Y Deep ZF. Improved DNA-binding prediction of C2H2-zinc-finger proteins by deep transfer learning. Bioinformatics. 2022;38(Suppl_2):ii62–ii7. 36124796 10.1093/bioinformatics/btac469

[11] Blake S Hemming I Heng JI. Structure-based approaches to classify the functional impact of ZBTB18 Missense variants in health and disease. ACS Chem Neurosci. 2021;12:979–89. 33621064 10.1021/acschemneuro.0c00758

[12] Xie B Khoyratty TE Abu-Shah E. The Zinc Finger Protein Zbtb18 represses expression of Class I phosphatidylinositol 3-Kinase subunits and inhibits plasma cell differentiation. J Immunol. 2021;206:1515–27. 33608456 10.4049/jimmunol.2000367 PMC7980533

[13] Zhang J Li Y Luo H. Analysis of clinical features and ZBTB18 gene variant in a child with autosomal dominant mental disorder type 22. Zhonghua yi xue yi chuan xue za zhi. 2022;39:293–6. 35315038 10.3760/cma.j.cn511374-20210630-00556

[14] van der Schoot V de Munnik S Venselaar H. Toward clinical and molecular understanding of pathogenic variants in the ZBTB18 gene. Mol Genet Genomic Med. 2018;6:393–400. 29573576 10.1002/mgg3.387 PMC6014478

[15] Meisler SL Gabrieli JDE. A large-scale investigation of white matter microstructural associations with reading ability. Neuroimage. 2022;249:118909. 35033675 10.1016/j.neuroimage.2022.118909 PMC8919267

[16] Ohtaka-Maruyama C Miwa A Kawano H. Spatial and temporal expression of RP58, a novel zinc finger transcriptional repressor, in mouse brain. J Comp Neurol. 2007;502:1098–108. 17447250 10.1002/cne.21350

[17] de Munnik SA Garcia-Minaur S Hoischen A. A de novo non-sense mutation in ZBTB18 in a patient with features of the 1q43q44 microdeletion syndrome. Eur J Hum Genet. 2014;22:844–6. 24193349 10.1038/ejhg.2013.249 PMC4023223

[18] McRae JF Clayton S Fitzgerald TW. Prevalence and architecture of de novo mutations in developmental disorders. Nature. 2017;542:433–8. 28135719 10.1038/nature21062 PMC6016744

[19] Ha M Huang X Li L. PKCalpha mediated by the PI3K/Akt-FOXA1 cascade facilitates cypermethrin-induced hyperthyroidism. Sci Total Environ. 2021;757:143727. 33250241 10.1016/j.scitotenv.2020.143727

